# New laparoscopic procedure for left-sided pancreatic cancer—artery-first approach laparoscopic RAMPS using 3D technique

**DOI:** 10.1186/s12957-017-1284-3

**Published:** 2017-12-02

**Authors:** Michihiro Yamamoto, Masazumi Zaima, Hidekazu Yamamoto, Hideki Harada, Junichiro Kawamura, Masahiro Yamada, Tekefumi Yazawa, Junya Kawasoe

**Affiliations:** Department of Surgery, Shiga Medical Center for Adults, 4-30 Moriyama 5-chome, Moriyama-city, Shiga-prefecture 524-8524 Japan

**Keywords:** Pancreatectomy, Pancreatic neoplasms, Laparoscopy

## Abstract

**Background:**

For left-sided pancreatic ductal adenocarcinoma (PDAC), radical antegrade modular pancreatosplenectomy (RAMPS) is a reasonable surgical approach for tumor-free margin resection and systemic lymph node clearance. In pancreaticoduodenectomy for PDAC, the superior mesenteric artery (SMA)-first approach (or the “artery-first approach”) has become the standard procedure. With improvements in laparoscopic instruments and techniques, some surgeons attempted to apply laparoscopic RAMPS (L-RAMPS) for carefully selected patients with left-sided PDAC. However, owing to several technical difficulties in this procedure, its application remains uncommon. Moreover, the artery-first approach in L-RAMPS has not been reported. Here, we developed the artery-first approach L-RAMPS for left-sided PDAC and have presented the same in this report.

**Case presentation:**

Between June 2014 and July 2015, 16 patients with left-sided PDAC were referred to our division for pancreatic resection. The following technique was used for performing L-RAMPS on 3 of the 16 patients (19%). Six trocars were placed. After opening the omental bursa, only the middle segment of the pancreas was initially separated from both the left renal vein and the SMA. We termed this procedure as the “artery-first approach using a dome-shaped dorsomedial dissection (3D) technique.” This 3D technique enabled the interruption of the entire arterial supply to the specimen while preserving the venous drainage through the splenic vein for preventing venous congestion. The technique also contributed to the early detection of no tumor infiltration into the SMA and the early determination of posterior dissection plane. After pancreatic neck transection, the splenic artery and vein were divided. Finally, the pancreatic tail and spleen were dissected in a right-to-left direction. All operations were completed without any intraoperative complications. The median blood loss and retrieved lymph node count were 75 mL and 37, respectively, which were superior to those reported by other previous studies on L-RAMPS. All resection margins were free of carcinoma. No severe postoperative complications were observed.

**Conclusions:**

The artery-first approach L-RAMPS using 3D technique is safe and feasible to perform. The significance of our proposed procedure is minimal blood loss and precise lymphadenectomy. Therefore, this novel technique may become the preferred treatment for left-sided PDAC in selected cases.

## Background

In pancreaticoduodenectomy (PD) for pancreatic ductal adenocarcinoma (PDAC), the superior mesenteric artery (SMA)-first approach (or the “artery-first approach”) has become the standard procedure [1–6]. This approach has the following advantages: (1) the early determination of resectability before performing irreversible surgical steps, (2) reduction in intraoperative blood loss by avoiding venous congestion of the specimen, (3) negative uncinate resection margin rate, (4) and adequate lymph node dissection along the SMA [1–8].

For left-sided PDAC, a novel procedure known as radical antegrade modular pancreatosplenectomy (RAMPS) was developed [9]. Briefly, dissection was performed from the right to the left direction in 1 of 2 posterior dissection planes, contributing to the achievement of negative posterior resection margins under plane view [9, 10]. In addition, the accompanying N1 lymph node dissection was based on an established anatomy of lymph node drainage for the left-sided pancreas [9, 11]. The long-term oncologic outcome with RAMPS for PDAC is acceptable, with a reported 5-year survival rate of 35% [12].

With advancements in laparoscopic instruments and techniques, some surgeons began using laparoscopic RAMPS (L-RAMPS) for carefully selected patients with left-sided PDAC [13–15]. However, this procedure remains uncommon due to several technical difficulties in performing it. Moreover, there have been no reports on the artery-first approach so far in L-RAMPS.

We developed a simple and novel technique for left-sided PDAC, termed as the artery-first approach L-RAMPS. Here, we have described the details and technical advantages of our procedure and evaluated the short-term clinical outcomes of the same.

## Case presentation

### Patients

From June 2014 to July 2015, 16 patients with PDAC around the body or at the tail of the pancreas were introduced to our division for pancreatic resection. Of these, 13 patients underwent open pancreatosplenectomy (RAMPS, *n* = 5; RAMPS with partial resection of the transverse colon, *n* = 1; RAMPS with partial liver resection, *n* = 2; RAMPS with cholecystectomy, *n* = 2; distal pancreatosplenectomy with celiac axis resection and portal vein resection and reconstruction, *n* = 3). The remaining three patients (19%) received the artery-first L-RAMPS using the proposed technique (Table 1). The indications before operation were as follows: (1) suspected PDAC around the body or at the tail of the pancreas, (2) no lymph node metastasis or no distant organ metastasis, (3) no infiltration to adjacent organs, (4) no infiltration to regional major vessels, excluding the splenic artery or vein, (5) no history of open laparotomy around the upper abdomen, (6) no history of severe pancreatitis, and (7) preference for laparoscopic treatment by the patient.

**Table 1:** Clinicopathologic characteristic outcomes for the three patients who underwent artery-first L-RAMPS for PDAC

Case	Age (years/sex)	BMI	Preop. pancreatitis	Additional organ excision	Degree of lymphadenectomy	Method for pancreatic transection	Curability	Op. time (min)	Bleeding (mL)	Retrieved lymph node count	Postop. complication	Postop. hospitalization (days)
1	81/M	22.5	no	T-colon	D2	Eschelon 60 green	R0	414	75	32	None	15
2	73/F	19.8	no	Left adrenal gland	D2	Electro-cautery	R0	454	10	58	DGE (grade 2) PF (grade B)	30
3	72/M	24.6	mild	no	D2	Eschelon 60 green	R0	421	130	37	None	14

*L-RAMPS* laparoscopic radical antegrade modular pancreatosplenectomy, *PDAC* pancreatic ductal adenocarcinoma, *BMI* body mass index, *op.* operative, *T-colon* transverse colon, *DGE* delayed gastric emptying, *PF* pancreatic fistula

This study protocol was approved by the Institutional Ethics Board of our institution. Written informed consent for the treatment was obtained from all patients.

### Surgical technique

**Fig. 1:**
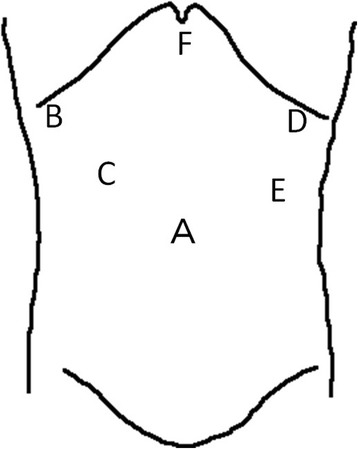
The patient is placed in the complete supine position with legs spread. For a, c, and d, 12-mm trocars are used. For b and e, 5-mm trocars are used. For f, a 3-mm trocar is used. A 30° rigid laparoscope is introduced through a

The patient was placed in a completely supine position, with legs spread out. The operating surgeon primarily stood on the right side of the patient, the assistant surgeon on the left, and the laparoscope operator between the legs of the patient. Six operating trocars were placed (Fig. 1). A 30° and 10-mm rigid laparoscope was introduced through the umbilical trocar. The operating surgeon directed the Harmonic ACE (Ethicon Endo-Surgery, Inc. Cincinnati, OH, USA) primarily with his right hand.

First, to expose the anterior surface of the pancreatic body, the omentum and the gastrocolic ligament were divided along the transverse colon and the greater curvature, respectively. The stomach was then retracted upwards by using a subxyphoid 3-mm grasper to ensure sufficient availability of surgical field around the suprapancreatic area.

**Fig. 2:**
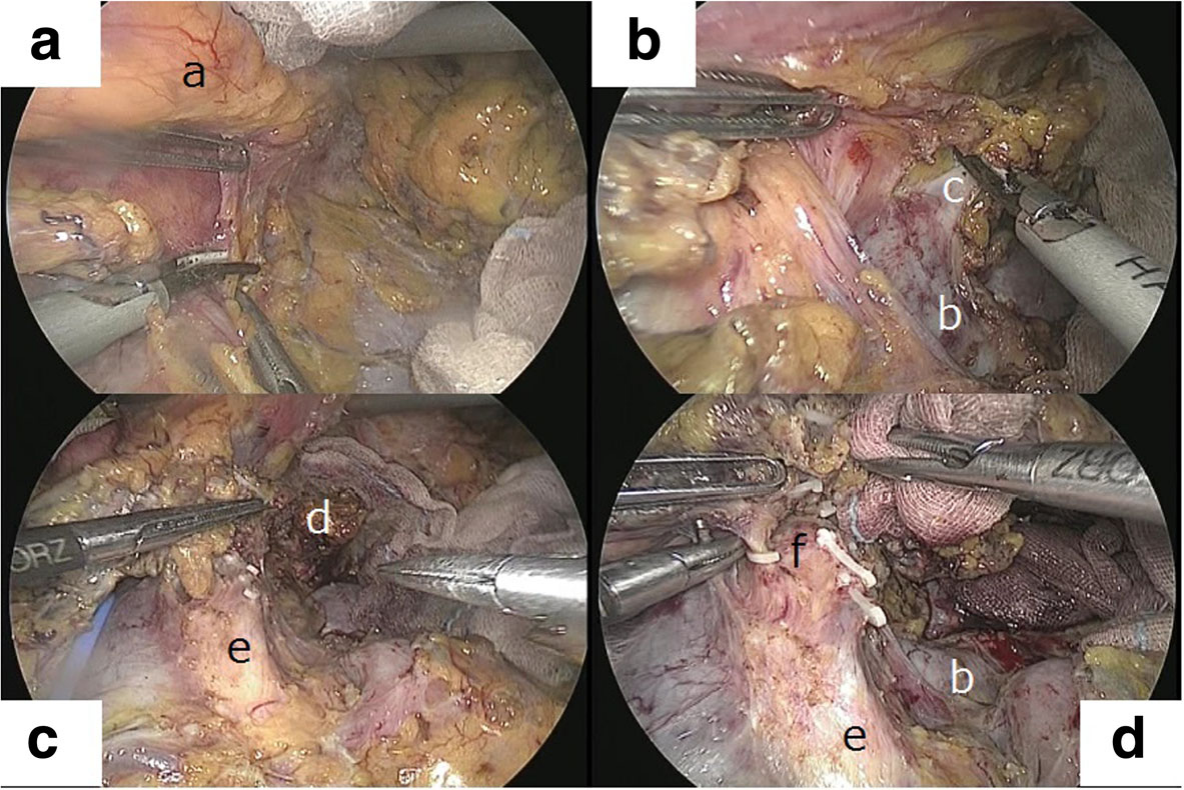
The outline of the artery-first approach using a dome-shaped dorsomedial dissection (3D) technique. **A** While both the pancreatic tail and spleen are kept attached in the subphrenic position, only the middle segment of the pancreas (a) is cranially mobilized toward the esophageal hiatus, resulting in obtaining a dome-shaped space behind the pancreatic body. **B** The left renal vein (b) is exposed to determine the dorsal dissection plane. The left adrenal vein (c) is simultaneously identified. **C** In the event of tumor infiltration to the anterior renal fascia, the left adrenal gland (d) is also excised with the pancreatic body. **D** The anterior surface of the superior mesenteric artery (SMA) (e), which can be identified below the pancreatic body, is cranially exposed. An arterial branching (f) to the pancreas from the SMA, known as the dorsal pancreatic artery, is divided

Second, to safely perform the artery-first approach with the “dome-shaped dorsomedial dissection” (3D) technique, only the middle segment of the pancreas was initially dissected off the left renal vein (Fig. 2). In particular, the inferior line of the left-sided pancreas was widely incised. While both the pancreatic tail and spleen were kept attached in the subphrenic position, only the middle segment of the pancreas was cranially mobilized toward the esophageal hiatus as a dome-shaped space (Fig. 2a). The mesocolon was pressed caudally with a piece of gauze by the assistant surgeon using a grasper, and the left renal vein was identified around the top of the Treitz ligament (Fig. 2b). The left renal vein was used as a landmark for determining the dorsal dissection plane, which included the anterior renal fascia [10]. If the tumor infiltrated to the anterior renal fascia or the left adrenal gland, the left adrenal gland was also dissected with the pancreatic body; this procedure is termed as “posterior RAMPS” [10] (Fig. 2c).

Following the 3D technique, the anterior surface of the SMA, which could be identified below the pancreatic body, was proximally exposed to its origin. The nerve plexus around the SMA should be dissected to secure R0 resection if required. With the detection of any tumor infiltration into the SMA wall, the operation was terminated. Arterial branches to the pancreas from the SMA, known as the dorsal pancreatic arteries (DPAs) [3], were divided under the laparoscopic view (Fig. 2d). Thanks to the 3D technique, surgeons could easily and safely separate the SMA and pancreas. Since the dome-shaped space at the left side of the SMA was already created (Fig. 2c, d), bleeding from a branch of the SMA could be easily controlled. After confirming that the anterior surface of the SMA was cancer-free, the superior mesenteric vein (SMV) was identified and exposed below the pancreas [3].

**Fig. 3:**
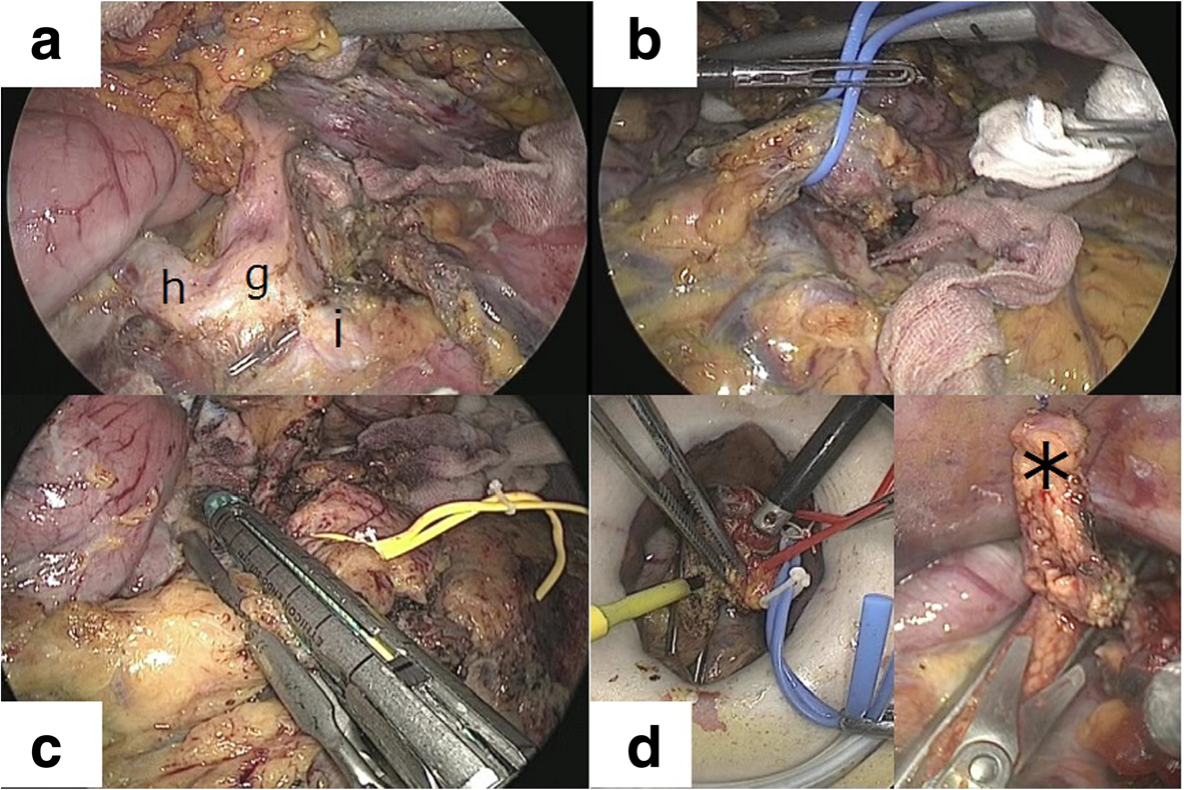
The suprapancreatic lymphadenectomy and the pancreatic neck transection. **A** En bloc lymphadenectomy with skeletonization of the celiac axis (g), common hepatic artery (h), left gastric artery, and splenic artery (i) is performed. **B** The pancreatic neck is encircled and taped. **C** The pancreatic neck is laparoscopically transected using a linear stapling device. **D** In the case of a tumor close to the neck, a 4-cm minilaparotomy is created immediately above the pancreatic neck for frozen pathologic examination of the stump and quick hemostasis. (* a sliced tissue sample of the stump)

Third, the entire arterial blood supply was interrupted to avoid venous congestion. The residual feeding arteries to the specimen were as follows: the splenic artery (Sp.A), posterior gastric artery, and transverse pancreatic artery through the pancreatic parenchyma. En bloc suprapancreatic lymphadenectomy with skeletonization of the common hepatic artery (CHA), left gastric artery, Sp.A, and celiac axis was performed (Fig. 3a). The left gastric vein was divided around the celiac axis. The gastropancreatic ligament as well as the posterior gastric arteries and veins were then divided along the gastric wall. If present, the DPA arising from the origin of the Sp.A or the CHA was divided. The portal vein (PV) was identified below the CHA, and the pancreatic neck was encircled and taped above the SMV (Fig. 3b). The neck, including the transverse pancreatic artery, was laparoscopically transected using a linear stapling device (Echelon Flex 60-mm; Ethicon Endo-Surgery, Inc. Cincinnati, OH, USA) (Fig. 3c).

For cases that required frozen pathologic examination of the pancreatic stump, a 4-cm minilaparotomy was created just above the pancreatic neck immediately before its transection. Under direct vision through the minilaparotomy, the neck was transected using a scalpel while being ventrally retracted with a tape (Fig. 3d). Tape retraction enabled safe cutting even through the minilaparotomy. Direct management through minilaparotomy allowed quick hemostasis on the pancreatic stump and secure oversewing of the main pancreatic duct (Fig. 3d), which would have been difficult without the use of the laparoscopic stapling device.

**Fig. 4:**
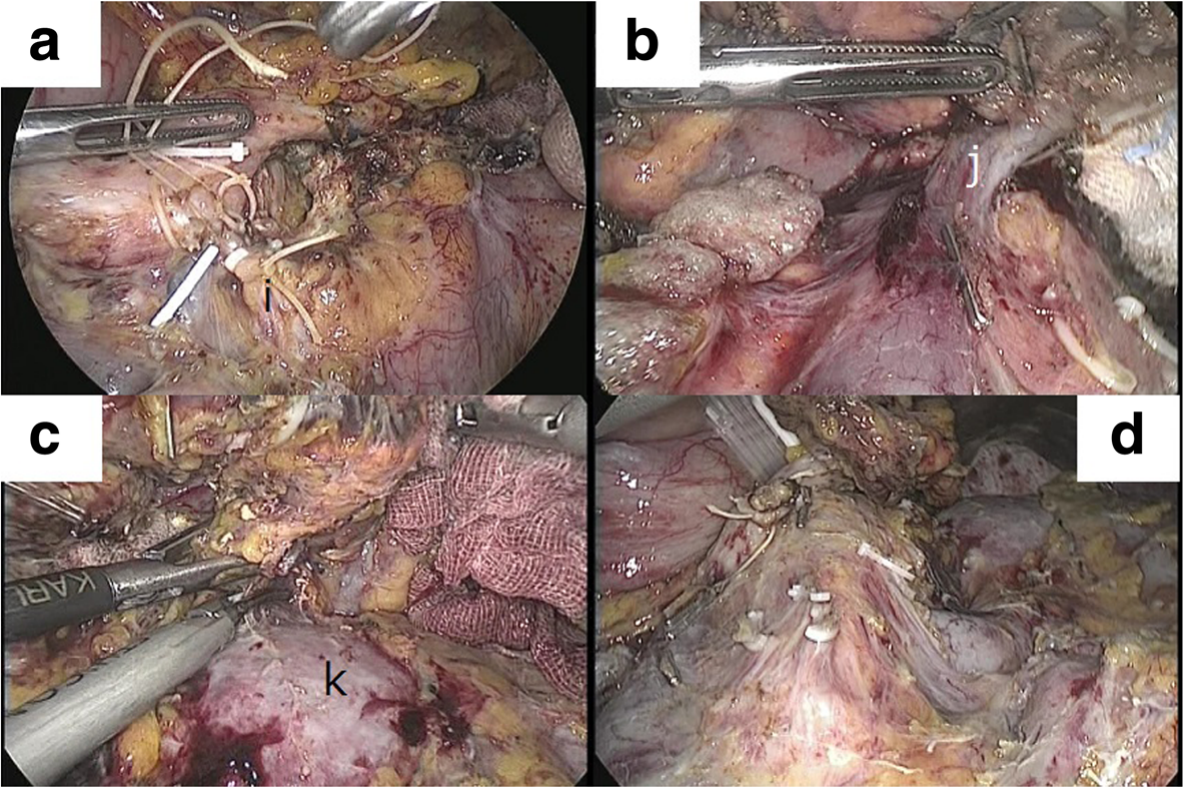
Intraoperative image in the final stage. **A** The origin of the splenic artery (i) is isolated and ligated in the suprapancreatic lesion without its division. Therefore, entire arterial blood supply to the specimen is interrupted. **B** The origin of the splenic vein (j), which is a main draining vessel, is isolated and divided. The splenic artery is then divided at its origin. **C** The pancreatic tail and the spleen are dissected off the left kidney (k) in a right-to-left fashion. **D** The artery-first laparoscopic radical antegrade modular pancreatosplenectomy (L-RAMPS) using a 3D technique is completed

The minilaparotomy was covered with the LAP PROTECTOR mini (Hakko Co., Nagano, Japan) combined with a surgical grove, and CO2 insufflation of the abdominal cavity was restarted. At the end of the third step, the origin of Sp.A, which could be easily isolated because the dorsal and caudal spaces were already created with the 3D technique and pancreatic transection, was laparoscopically ligated without division (Fig. 4a). At this point, the entire arterial blood supply to the specimen was interrupted while venous drainage through the splenic vein (Sp.V) was maintained.

Fourth, the origin of the Sp.V was clipped and divided (Fig. 4b) so that the operative field around the celiac axis was widened. Therefore, the Sp.A was then securely divided at its origin. Finally, the pancreatic tail and the spleen were dissected with the surrounding soft tissues in a right-to-left direction (Fig. 4c). Since the dome-shaped retropancreatic space was already created, the dissection plane was easily selected under the laparoscopic view. The specimen was introduced into an endopouch and retrieved through the umbilical or the previous 4-cm minilaparotomy, with which the artery-first L-RAMPS using a 3D technique was completed (Fig. 4d).

## Results

The artery-first L-RAMPS using a 3D technique was potentially applicable to 8 of the 16 cases (50%) preoperatively based on our indication criteria pertaining to tumor-related variables and physical conditions of the patient. However, five out of the eight patients opted for open surgery. In all, the artery-first L-RAMPS using a 3D technique was successfully performed in three patients, without any intraoperative complications. Table 1 shows the clinicopathologic characteristics and operative data. Case 1 required combined resection of the transverse colon due to tumor infiltration. Case 2 underwent laparoscopy-assisted operation for frozen examination of the pancreatic stump. The patient also required combined resection of the left adrenal gland due to tumor invasion. The median operation time was 421 min (range, 414–454 min), intraoperative blood loss was 75 mL (range, 10–130 mL), and the retrieved lymph nodes count was 37 (range, 32–58). The pathologic diagnosis was invasive ductal adenocarcinoma in all cases. Microscopically, all surgical margins were free of carcinoma. Lymph node metastasis was detected in cases 1 and 2. Only case 2 had postoperative complications, which were grade B pancreatic stump fistula (according to definition of the International Study Group of Postoperative Pancreatic Fistula) and grade 2 delayed gastric emptying (according to the Common Terminology Criteria for Adverse Events, version 4.0). Both complications were treated with conservative management.

## Discussion and conclusions

Our new laparoscopic procedure for left-sided PDAC, artery-first L-RAMPS using a 3D technique, was safe and feasible to perform. This procedure is advantageous as it allowed minimal intraoperative blood loss and precise lymphadenectomy.

L-RAMPS reported previously [13–15] had several technical drawbacks. First, these L-RAMPS procedures [13–15] were not described as artery-first approaches. During pancreatic cancer surgery, tumor infiltration to the SMA should be detected before irreversible surgical steps need to be taken, such as pancreatic transection or the division of the splenic vessels. In fact, in the PD field, the artery-first approach is fast becoming one of the standard procedures [1–6].

The second drawback in these studies is that the L-RAMPS procedures were not based on the prevention of venous congestion of the specimen. The Sp.V, which is the main draining vessel, is generally a hindrance in the assessment of both the origin of the Sp.A and the anterior surface of the SMA even after pancreatic neck transection, particularly when using the laparoscopic approach. Consequently, interruption of all arterial blood flow before division of the Sp.V is technically demanding. Venous congestion of the specimen poses a risk of increasing intraoperative bleeding, which may prevent meticulous lymphadenectomy. Indeed, previous reports have indicated excessive blood loss and fewer counts of retrieved lymph nodes [13–15].

The third drawback of the previously reported L-RAMPS procedures is that a dry operative field was difficult to maintain laparoscopically. Since the pancreas is one of the retroperitoneal organs, the blood and lymphatic fluid are prone to collect around the pancreas due to gravity, thereby interfering with lymphadenectomy.

To overcome these technical drawbacks, we developed artery-first L-RAMPS method using a 3D technique. Our procedure has four technical advantages. First, the 3D technique enabled interruption of the entire arterial blood supply to the specimen while preserving the Sp.V, thereby preventing venous congestion of the specimen. Early division of the DPAs from the SMA was also easier after dome-shaped dorsomedial pancreatic dissection (Fig. 2d). Intraoperative venous congestion of the specimen was not observed in any of the cases. As a result, the median amount of intraoperative blood loss was 75 mL (range, 10–130 mL), which is much lesser than 364 mL (range, 0–3000 mL), as reported by other laparoscopic studies for left-sided PDAC [13, 16–20].

The second advantage was that the 3D technique enabled early detection of tumor infiltration into the SMA or to the left adrenal gland before pancreatic transection. With this, surgeons could decide between early termination of the operation and performing a combined resection of the left adrenal gland. In the original open RAMPS procedure [9, 10, 12], the exposure of the SMA and the detection of the posterior dissection plane around the left kidney occurred after pancreatic neck transection, which is an irreversible surgical step. Indeed, combined adrenal gland resection was immediately decided during the 3D technique in case 3.

The third advantage of L-RAMPS using a 3D technique was that the spleen remained attached in the subphrenic area during most of the operation period. In general, mobilization of the spleen has a risk of injury to its capsule, which may result in uncontrollable massive bleeding that can interrupt the operative procedure. Our present technique did not cause any splenic injury in any of the patients. In addition, the dome-shaped dorsomedial pancreatic space was easily maintained with one hand by the assistant surgeon (Fig. 2), in contrast to the wide detachment of the distal pancreas and spleen. Owing to the 3D technique, the assistant surgeon could use his other hand for retraction of the transverse mesocolon.

The fourth advantage was that, in our technique, the blood and lymphatic fluid pooled in the dome-shaped dorsal space due to gravity. Therefore, we succeeded in obtaining a dry and clear operative field and could perform meticulous lymphadenectomy (Figs. 3a, b and 4a). In our series, the median retrieved lymph node count was 37 (range, 32–58), which is greater than 14 (range, 1–43) in other laparoscopic reports on L-RAMPS in high volume centers [13, 16–20], resulting in oncologic acceptability. We believe that these four technical advantages led to the successful outcomes in our study.

In the case of a tumor close to the pancreatic neck, as in case 2, a minilaparotomy just above the pancreatic neck was useful for frozen pathologic examination of the pancreatic stump. Moreover, direct management through the minilaparotomy allowed quick hemostasis on the stump as well as the secure closure of the main pancreatic duct (Fig. 3d).

When the exposure of the left renal vein was difficult during the procedure, utilizing the Treitz ligament approach appeared to be helpful. In detail, after the peritoneum around the Treitz ligament is opened, the third duodenal portion can be cranially mobilized from the abdominal aorta to reach the left renal vein [13, 21].

In this study, no intraoperative complications or massive bleeding were noted. However, our median operation time was longer than that reported otherwise due to the learning curve, which could probably be because we had never performed laparoscopic pancreatectomy for PDAC before. Moreover, cases 1 and 2 required combined resection of the transverse colon and the left adrenal gland due to direct infiltration of the tumor, respectively. Case 3 had preoperative pancreatitis. As our present technique is simple, experienced laparoscopic surgeons can probably accomplish it in a shorten span of operation time. The postoperative hospitalization seemed long in our case, probably because of the unique insurance system in Japan, wherein the cost of hospitalization is relatively low.

This study had some limitations. We included a small number of subjects, patient selection was limited, and only short-term outcomes were evaluated. We hope to evaluate the long-term oncologic outcomes for this procedure as well as apply this technique to more patients in the future. Judging from the clinical outcomes in this study, our technique has significant scope to improve minimally invasive surgical treatment for left-sided PDAC.

In conclusion, artery-first L-RAMPS using a 3D technique for left-sided PDAC is a safe and feasible procedure. This procedure may play an important role in reducing intraoperative blood loss and in ensuring high precision of lymphadenectomy. With several theoretical advantages, we believe this novel technique may become the preferred treatment for left-sided PDAC in selected cases.

## Abbreviations

3D: Dome-shaped dorsomedial dissection
CHA: Common hepatic artery
DPA: Dorsal pancreatic artery
L-RAMPS: Laparoscopic RAMPS
PD: Pancreaticoduodenectomy
PDAC: Pancreatic ductal adenocarcinoma
PV: Portal vein
RAMPS: Radical antegrade modular pancreatosplenectomy
SMA: Superior mesenteric artery
SMV: Superior mesenteric vein
Sp.A: Splenic artery
Sp.V: Splenic vein
